# Elevated double-strand break repair protein RAD50 predicts poor prognosis in hepatitis B virus-related hepatocellular carcinoma: A study based on Chinese high-risk cohorts

**DOI:** 10.7150/jca.46703

**Published:** 2020-08-14

**Authors:** Wangrui Liu, Wenhao Xu, Yuyan Chen, Liugen Gu, Xiaolei Sun, Yuanyuan Qu, Hailiang Zhang, Xiaojuan Liu, Haineng Huang

**Affiliations:** 1 Department of Neurosurgery, Affiliated Hospital of Youjiang Medical University for Nationalities, Guangxi, 533000, China.; 2 Clinical College of Youjiang Medical University for Nationalities, Baise, Guangxi, 533000, China.; 3 Department of Urology, Fudan University Shanghai Cancer Center, Shanghai 200032, China.; 4 Department of Oncology, Shanghai Medical College, Fudan University, Shanghai 20032, China.; 5 Department of Gastrointestinal Surgery, Nantong University Affiliated Hospital, Nantong, Jiangsu, 226001, China.; 6 Gastroenterology Department, Second affiliated Hospital of Nantong University, Nantong, Jiangsu, 226001, China.; 7 Department of Pathogenic Biology, Medical College, Nantong University, Nantong, Jiangsu, 226001, China.

**Keywords:** RAD50, hepatocellular carcinoma, DNA repair, multiple cohorts, prognosis

## Abstract

**Objective:** Increasing evidence indicates that RAD50, which is involved in the repair process of DNA double-strand break (DSB), is also involved in cancer outcomes. However, its role in hepatitis B virus (HBV)-related hepatocellular carcinoma (HCC) remains unclear. This study was designed to investigate the expression of RAD50 and its prognostic value in HBV-related HCC patients.

**Methods:** 107 and 100 patients with HBV-related HCC from the Affiliated Hospital of Youjiang Medical University of Nationalities (AHYMUN) and the Affiliated Hospital of Nantong University (AHNU), respectively, were enrolled in the study. The distribution of the categorical clinical-pathological data and the levels of RAD50 expression were compared with a χ^2^ test. Immunohistochemistry (IHC) staining of RAD50 was performed. A partial likelihood test based on univariate and multivariate Cox regression analysis was developed to address the influence of independent factors on disease-free survival (DFS) and overall survival (OS). The Oncomine online database was used to analyse and validate the differential expression of RAD50. The Kaplan-Meier method and a log-rank test were performed to assess the influence of RAD50 on survival at different levels.

**Results:** RAD50 was highly expressed in HCC tissues compared to normal tissues and was significantly correlated with OS in the Cancer Genome Atlas (TCGA) cohort. The validation analysis indicated that significantly increased levels of RAD50 were expressed in HCC tissues in the two independent cohorts. In addition, HCC patients with elevated RAD50 expression levels showed poor OS and DFS in the AHYMUN cohort and decreased OS and DFS in the AHNTU cohort.

**Conclusion:** In conclusion, our study reveals that elevated RAD50 expression is significantly correlated with cancer progression and poor survival in HBV-related HCC patients. These data suggest that RAD50 may act as an oncogene and may serve as a promising target for the therapy of HBV-related HCC patients.

## Introduction

In China, the incidence and mortality of primary hepatocellular carcinoma (HCC) is the second highest of all malignant cancers 1. In addition, in China, because the incidence of hepatitis B virus (HBV) infection is high, HBV accounts for at least 80% of HCC cases. In the Guangxi Zhuang Autonomous Region (Guangxi province) 2 and Qidong in Nantong Prefecture (Jiangsu province) 3, HBV infection is very common, leading to a high incidence of HCC 4, 5. Although multiple markers, such as alpha fetoprotein (AFP), have been widely used in clinical practice, the accuracy and sensitivity of diagnosis and targeted therapy need to be improved. Therefore, to reduce the cost of treatment for patients with HBV-related HCC and to improve their quality of life, it is important to understand the underlying molecular mechanisms and apply them to the development of targeted treatments.

The DNA repair component protein Rad50 (RAD50) is a component of the MRE11-RAD50-NBS1 (MRN) complex, which is a heterotrimer composed of meiotic recombination 11 (MRE11), RAD50, and Nijmegen fracture syndrome 1 (NBS1), participating in the DNA damage response (DDR). Recent studies have found that DDR may be related to the occurrence and development of cancers. Defective mitosis-linked DNA damage response and chromosomal instability facilitate the progression of liver cancer 6. In addition, the mutation of RAD50 has an adverse effect on the prognosis of prostate cancer patients 7. Increased RAD50 expression has been found in multiple tumours, including breast cancer, ovarian cancer, lung cancer and rectal cancer 8-11. For example, RAD50 increases in colorectal cancer patients have been shown to be positively related to tumour development and prognosis 12. It is speculated that the high expression of RAD50 leads to cell instability, which increases the tendency toward DNA damage accumulation and malignant transformation. Following HBV infection, RAD50 expression in hepatocytes has been shown to be increased 13.

To investigate the expression and prognostic value of RAD50 in HBV-related HCC, we recruited 207 HBV-related HCC patients from two centres. The prognostic role of RAD50 in HCC patients was validated and functionally annotated *in silico*. We speculate that RAD50 is associated with poor prognosis and may serve as a potential therapeutic target for HBV-related HCC.

## Materials and Methods

### Patients and Variables

In this study, a total of 107 HCC patients were recruited from the Affiliated Hospital of Youjiang Medical University of Nationalities (AHYMUM) (Youjiang, Guangxi, China) between April 2008 and October 2018, and 100 HCC patients were also recruited from the Affiliated Hospital of Nantong University (AHNTU) (Nantong, Jiangsu, China) for analyses between July 2013 and May 2017. The inclusion criteria consisted of the following: (1) HCC diagnosed by pathology (surgery or biopsy), typical dynamic imaging studies, and AFP serology according to the American Association for the Study of Liver Diseases (AASLD) guidelines 17; (2) chronic hepatitis B virus infection [hepatitis B surface antigen (+)]; (3) no previous oncological treatment or liver resection; (4) no tumour invasion in the branches or trunk of the portal vein or hepatic veins according to MRI; (5) no other diffuse liver disease, including primary sclerosing cholangitis or primary biliary cirrhosis. The clinical and pathological parameters in the two cohorts, including age at surgery, gender, tumour size, American Joint Committee on Cancer (AJCC) stage, primary tumour lesion, the degree of pathological nuclear differential expression, Okuda score, albumin level, bilirubin level, presence of liver cirrhosis, and the degrees of microvascular invasion and capsular invasion are summarized in Table 1. Tissue samples, including HCC and adjacent normal tissues, were collected during surgery and were available from the AHYMUM and AHNTU tissue banks. All study designs and test procedures were performed in accordance with the Helsinki Declaration II. Ethical approval and consent to participate in the current study were obtained from the ethics committee.

### Immunohistochemistry (IHC)

Immunostaining of RAD50 was performed using a mouse monoclonal anti-RAD50 antibody (1:1000, #Ab89, Abcam, USA). Positive or negative staining of a specific protein on one formalin-fixed paraffin-embedded (FFPE) slide was independently assessed by two experienced pathologists and determined according to the following descriptions. The staining intensity level was graded from 0 to 3. Samples with no staining or weak, median or strong staining were graded at the level of 0, 1, 2 or 3, respectively. The staining extent ranged from 0 to 4 based on the percentage of coverage of the immunoreactive tumour cells (0%, 1-25%, 26-50%, 51-75%, or 76-100%, respectively). The overall IHC score, which was graded on a scale from 0 to 12, was generated by multiplying the staining intensity and extent scores. Negative staining was indicated by a grade from 0 to 3 and positive staining was indicated by a grade from 4 to 12 for each sample. All samples were classified into the tumour or normal group to confirm the differential expression of RAD50.

### Statistical analysis

To determine the association of different RAD50 mRNA expression levels with clinicopathological characteristics, a χ^2^-test was performed to compare the distribution of the categorical data between groups. A scatter plot was utilized to represent the differential expression of RAD50 in normal and HCC tissues. The primary end point was overall survival (OS) for patients who survived for a specific period of time, which was determined according to the length of time from the date of surgery to the date of death or date of last follow-up. Disease-free survival (DFS), as the secondary end point, was defined as the length of time from the initiation of curative treatment when no disease could be detected until the date of progression, the initiation of second-line treatment or the date of death, which ever occurred first. The follow-up duration was estimated using the Kaplan-Meier method with a 95% confidence interval (CI) and a log-rank test with separate curves. The hazard ratios were derived from Cox proportional hazard regression models based on a high-versus-low comparison to identify the independent predictors. Univariate and multivariate Cox regression models were independently analysed to evaluate the influence of the confounding covariates, including age at surgery, gender, tumour size, AJCC stage, primary tumour site, the degree of pathological nuclear differential expression, Okuda score, albumin level, bilirubin level, presence of liver cirrhosis, presence of microvascular invasion, presence of capsular invasion, and RAD50 expression, on survival. Statistical analyses were performed with SPSS software (version 23.0, SPSS Inc., Chicago, IL, USA). All hypothetical tests were two-sided, and P-values less than 0.05 were considered significant for all tests.

### Determination of differential RAD50 expression based on Oncomine datasets

RAD50 expression profiles in hepatocellular tumour and normal tissue were analysed and displayed using the Oncomine online database (http://www.oncomine.com). A dataset including the known gene expression patterns in human HCC were included to validate the mRNA expression of RAD50 in the two groups. An unpaired t test was used to analyse the significant differences.

### Data processing of Gene Set Enrichment Analysis (GSEA)

The Search Tool for the Retrieval of Interacting Genes (STRING; http://string-db.org) (version 10.0) online database was used to predict the protein-protein interaction (PPI) network for RAD50. Any interaction with a combined score > 0.4 was considered statistically significant. The significant genes were included in functional and pathway enrichment analyses in a bubble chart using DAVID bioinformatics resources, which indicated the most significant signalling pathways according to Gene Ontology (GO) and Kyoto Encyclopedia of Genes and Genomes (KEGG) analysis. (GO) enrichment analysis, including biological processes (BP), cellular components (CC), and molecular functions (MF). Datasets from TCGA database were analysed with the GSEA method using the category version 2.10.1 package in R. For each separate analysis, the Student's t-test statistical score was obtained for each of the consistent pathways, and the mean differential expression of each gene was calculated. A permutation test was performed 1000 times to identify the pathways with significant changes. The adjusted P values (adj. P), which were obtained by default using the Benjamini and Hochberg (BH) false discovery rate (FDR) method, were utilized to correct for the occurrence of false positive results. Significantly related genes were defined by an adj. P less than 0.01 and an FDR less than 0.25.

In this study, the staining intensity level was graded from 0 to 3 (0-25%, 26-50%, 51-75%, or 76-100%). Samples with no staining or weak, median or strong staining were graded at the level of 0, 1, 2 or 3, respectively. The staining extent ranged from 0 to 4 based on the percentage of coverage of the immunoreactive tumour cells (0%, 1-25%, 26-50%, 51-75%, or 76-100%, respectively). The overall IHC score, which was graded on a scale from 0 to 12, was generated by multiplying the staining intensity and extent scores. Negative staining was indicated by a grade from 0 to 3 and positive staining was indicated by a grade from 4 to 12 for each sample. The different intensity of RAD50 expression ranging from 0-25%, 26-50%, 51-75%, and 76-100% was shown in **Fig. S1**.

## Results

In this study, the research was conducted in three steps. In the first step, the differential expression of RAD50 in normal and liver tissues was analysed; in the second step, RAD50 expression in HCC patients was assessed as an indicator of progression and prognosis; in the third step, the differential expression of RAD50 was validated in the Oncomine datasets, and GSEA was used to identify the relationships between significant genes and the involved signalling pathways.

### Prognostic role of RAD50 in HCC based on TCGA

As shown in **Table 1**, in the AHYMUM cohort, increased RAD50 expression was significantly correlated with decreased age (*p* = 0.021), advanced AJCC stage (*p* = 0.001), the presence of multiple primary tumour lesions (*p* = 0.001), the presence of micro vascular invasion (*p* = 0.028), the presence of capsular invasion (*p* = 0.015), higher HBV DNA level (*p* = 0.004), increased Okuda score (*p* = 0.009) and increased Child-Pugh Stage (*p* = 0.025). In the AHNTU cohort, increased RAD50 expression was significantly associated with larger tumour size (*p* < 0.001), advanced AJCC stage (*p* = 0.003), the presence of multiple primary tumour lesions (*p* = 0.010), the degree of pathological nuclear differential expression (*p* = 0.005), the presence of microvascular invasion (*p* = 0.001), the presence of capsular invasion (*p* = 0.003), and higher Okuda score (*p* = 0.008). A chi-square test showed that the baseline data were balanced in terms of the distribution of the categorical data, including gender, albumin level, bilirubin level and the presence of liver cirrhosis (*p* > 0.05).

**Figure 1:**
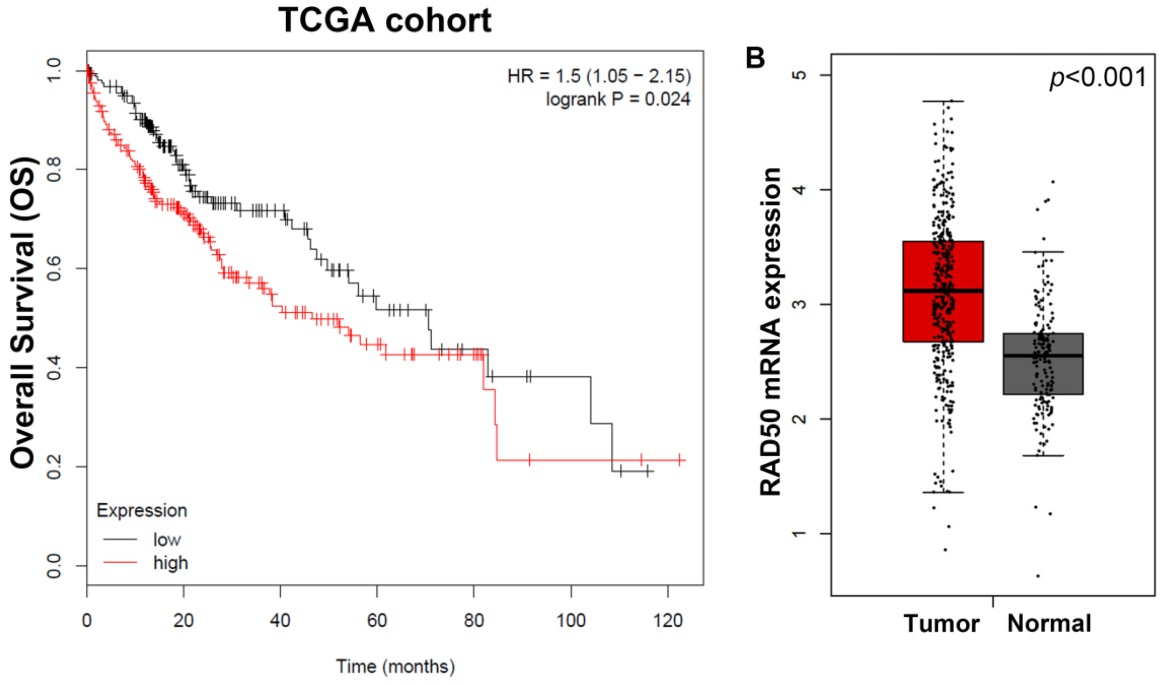
** A.** Kaplan-Meier survival analyses of different RAD50 expression groups based on OS in 207 patients with hepatocellular carcinoma from the TCGA database; **B.** Differential transcriptomic expression of RAD50 in tumour and normal tissues in the TCGA cohorts.

As shown in **Fig. 1A**, the Kaplan-Meier survival analyses of different RAD50 expression groups based on OS in 207 patients with HCC from the TCGA database, patients with high RAD50 expression have significantly lower OS than patients with low expression. Moreover, in the TCGA cohorts, the expression of RAD50 in tumour tissues is significantly higher than that in normal tissues (**Fig. 1B**).

### External validation of differential expressed RAD50 in multiply cohorts

**Figure 2:**
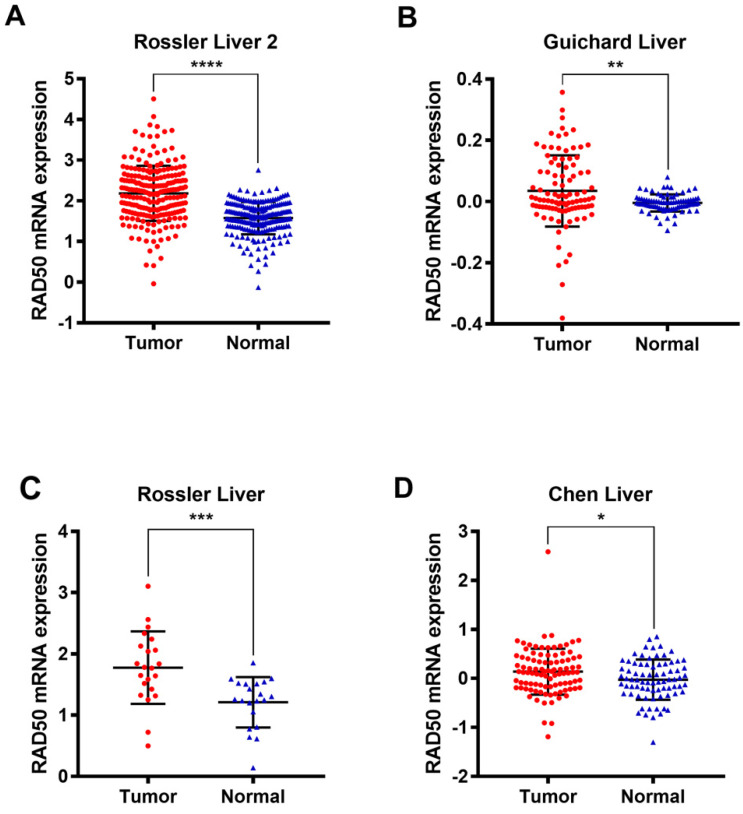
Analysis of expression in hepatocellular tumour vs. normal tissues based on data from the Oncomine database. **A.** Roessler, et al., A unique metastasis gene signature enables prediction of tumour relapse in early-stage hepatocellular carcinoma patients, Cancer Res, 2010. **B.** Guichard C, et al., Integrated analysis of somatic mutations and focal copy-number changes identifies key genes and pathways in hepatocellular carcinoma, Nature Genetics, 2012. **C.** Roessler, et al., A unique metastasis gene signature enables prediction of tumor relapse in early-stage hepatocellular carcinoma patients, Cancer Res, 2010. **D.** Chen X, et al., Gene expression patterns in human liver cancers, MolBiol Cell, 2002.

Four datasets (Rossler Liver 2 14, Guichard Liver 15, Rossler Liver 14, and Chen Liver 16) from the Oncomine database were analysed to validate the differential expression of RAD50 in HCC tumour and normal tissues (**Fig. 2**).

**Figure 3:**
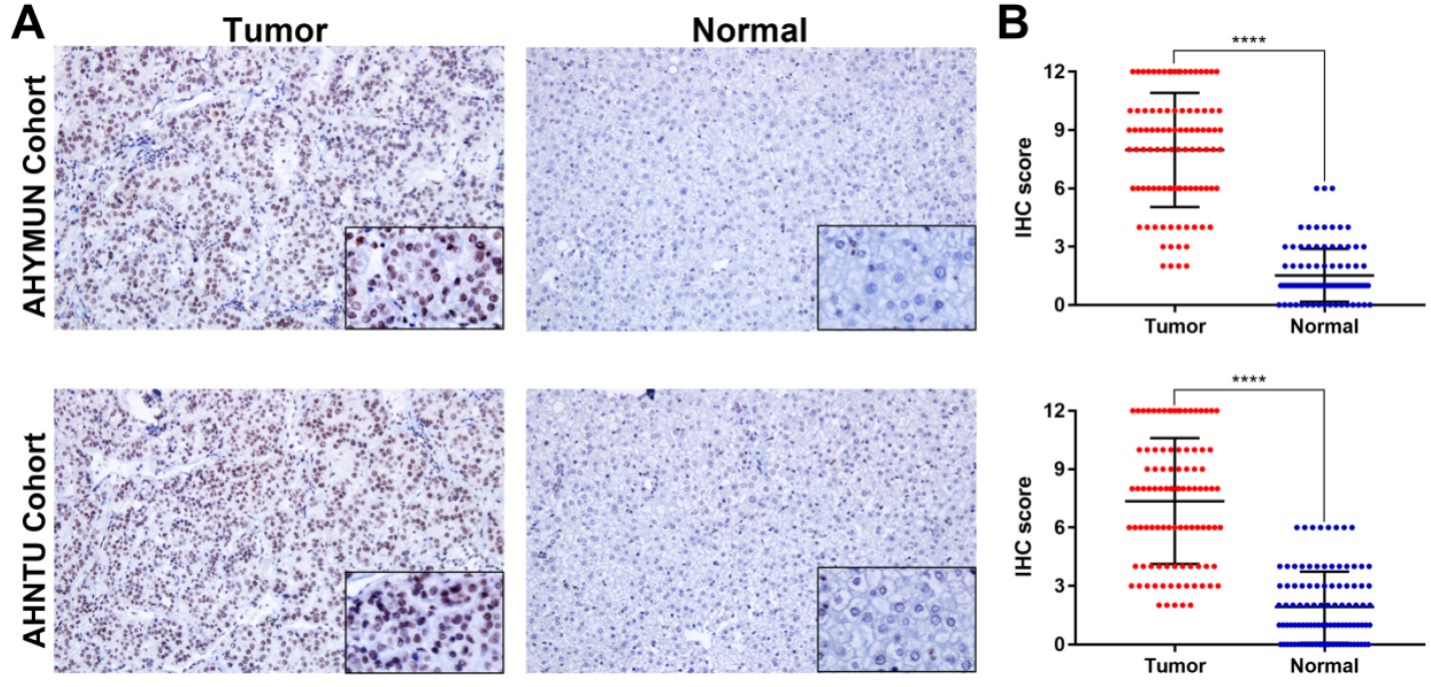
IHC staining and scatter plot analysis of hepatocellular carcinoma and normal tissues of patients in the AHYMUN and AHNTU cohorts. **A.** IHC staining of hepatocellular carcinoma and normal tissues from patients in the AHYMUN and AHNTU cohorts; **B.** Scatter plot showing the IHC scores derived from normal and tumour tissues (*p* < 0.001) from different cohorts.

To analyse the RAD50 expression, IHC was performed to reveal the staining distribution in tumour and normal tissues (**Fig. 3A**). The scatter plot of the IHC scores revealed that RAD50 expression was significantly elevated in HCC tissues in the AHYMUM (*p* < 0.001) and AHNTU (*p* < 0.001) cohorts (**Fig. 3B**).

### Cox regression analyses

**Figure 4:**
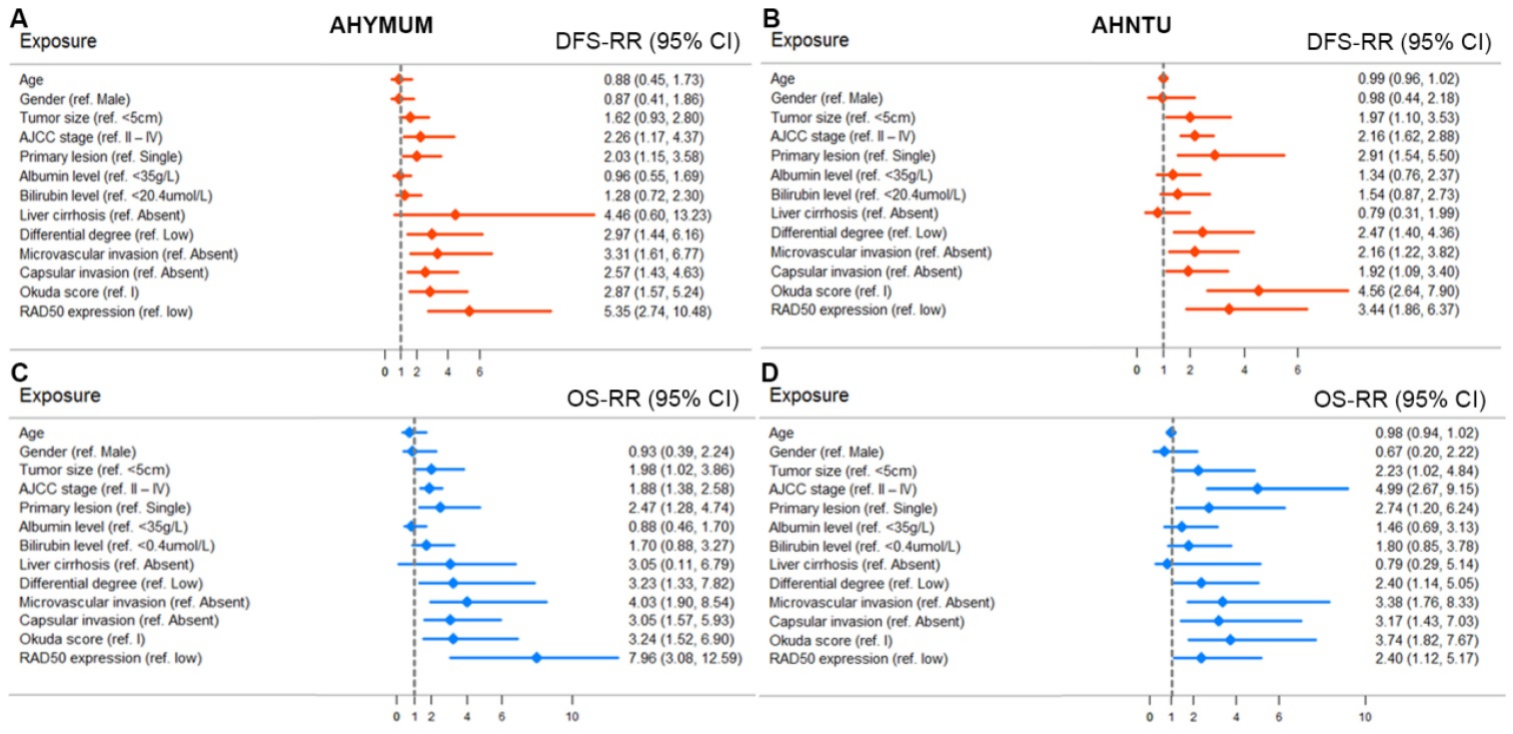
Forest plots were used to visualize the univariate Cox regression analysis of DFS and OS in the AHYMUM and AHNTU cohorts.

The univariate Cox regression analysis, as shown in the forest plots, revealed that age, gender, albumin level, bilirubin level and liver cirrhosis were not significantly associated with DFS in the patients form both AHYMUN (**Fig. 4A**) and AHNTU (**Fig. 4B**) cohorts. Meanwhile, tumour size was not an independent predictor of DFS in the AHYMUN cohort. In the multivariate models, the Okuda score (ref. I) was significantly associated with DFS in HCC patients, indicating the good representativeness of the population in the AHYMUN (HR: 1.914, *p* = 0.045) and AHNTU (HR: 3.084, *p* = 0.001) cohorts. More importantly, the subgroup analysis of RAD50 expression showed that RAD50 amplification was significantly correlated with DFS in the AHYMUN (HR: 4.612, *p* < 0.001) and AHNTU (HR: 2.660, *p* = 0.006) cohorts. Additionally, in the AHNTU cohort, a number of primary lesions was significantly correlated with poor DFS (HR: 2.563; *p* = 0.007) according to the multivariate model used for the Cox regression analyses (**Table 2**).

In **Fig. 4C and 4D,** several parameters, including age, gender, albumin level, bilirubin level and the presence of liver cirrhosis, were not significantly associated with OS in the two cohorts. As shown in **Table 3**, the multivariate Cox analysis indicated that the Okuda score (ref. I) was associated with OS in both the AHYMUN (HR: 2.329, *p* = 0.031) and AHNTU (HR: 2.819, *p* = 0.030) cohorts. RAD50 expression (ref. low) was associated with OS in AHYMUM patients (HR: 6.807; *p* < 0.001) but was not markedly correlated with OS in HCC patients from the AHNTU (HR: 1.735; *p* = 0.195) cohort. The other factors, including tumour size (ref. < 5 cm), the presence of multiple primary lesions (ref. single), the degree of pathological nuclear differential expression (ref. low), the presence of microvascular invasion (ref. absent) and the presence of capsular invasion (ref. absent), were not identified as prognostic indicators of OS (*p* > 0.05).

**Figure 5:**
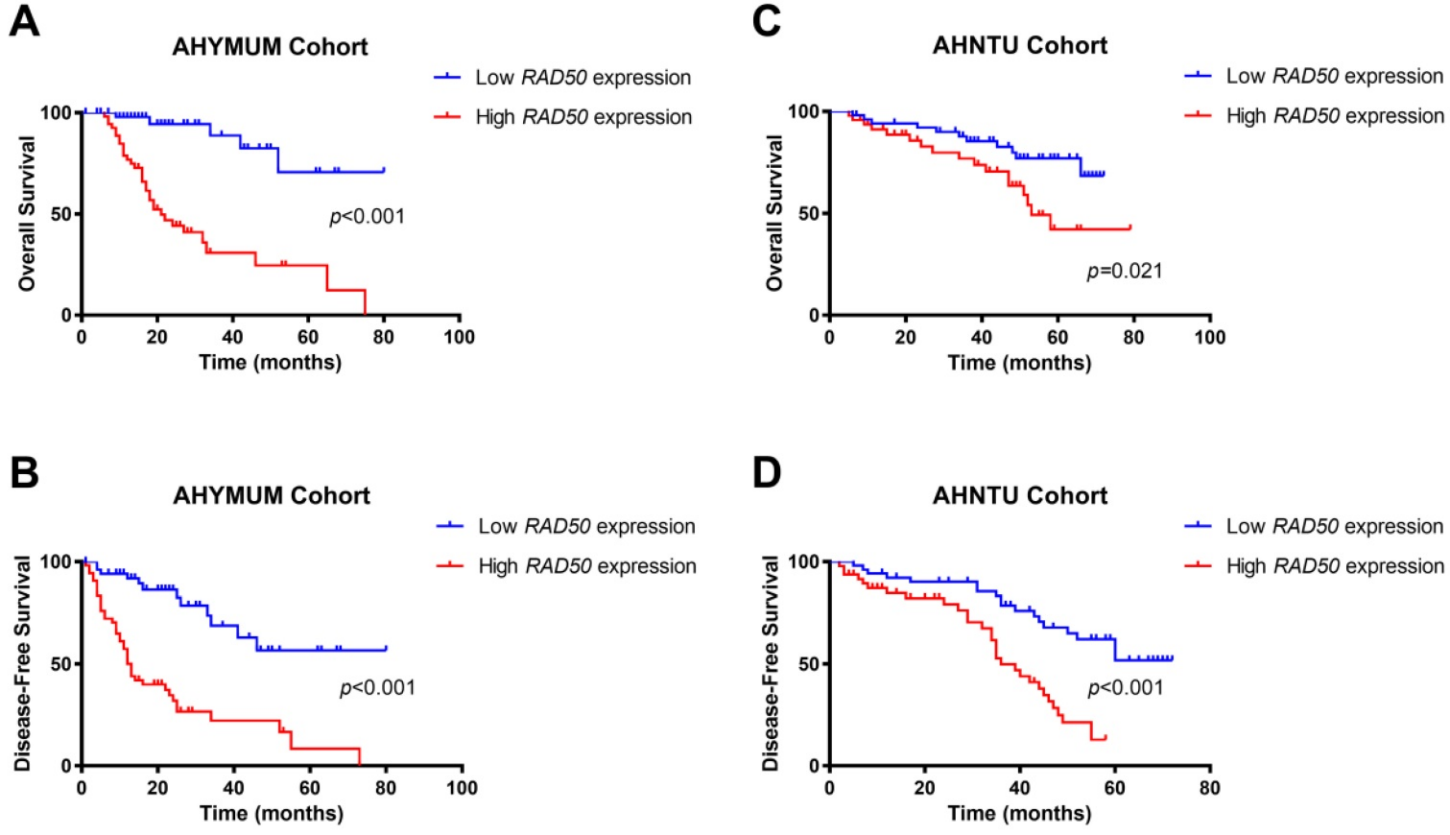
Kaplan-Meier survival analyses of different RAD50 expression groups based on OS and DFS in 107 patients with HBV-related HCC from the AHYMUM cohort (**A & B**) and in 100 patients from the AHNTU cohort (**C & D**).

The survival curves suggested that HCC patients in the AHYMUN cohort with elevated RAD50 expression levels showed poor OS (*p* < 0.001) and poor DFS (*p* < 0.001) (**Fig. 5A and 5B**). In addition, in the AHNTU cohort, increased RAD50 expression was significantly associated with decreased OS (*p* = 0.021) and DFS (*p* < 0.001) (**Fig. 5C and 5D**).

### Expression validation and GSEA

**Figure 6:**
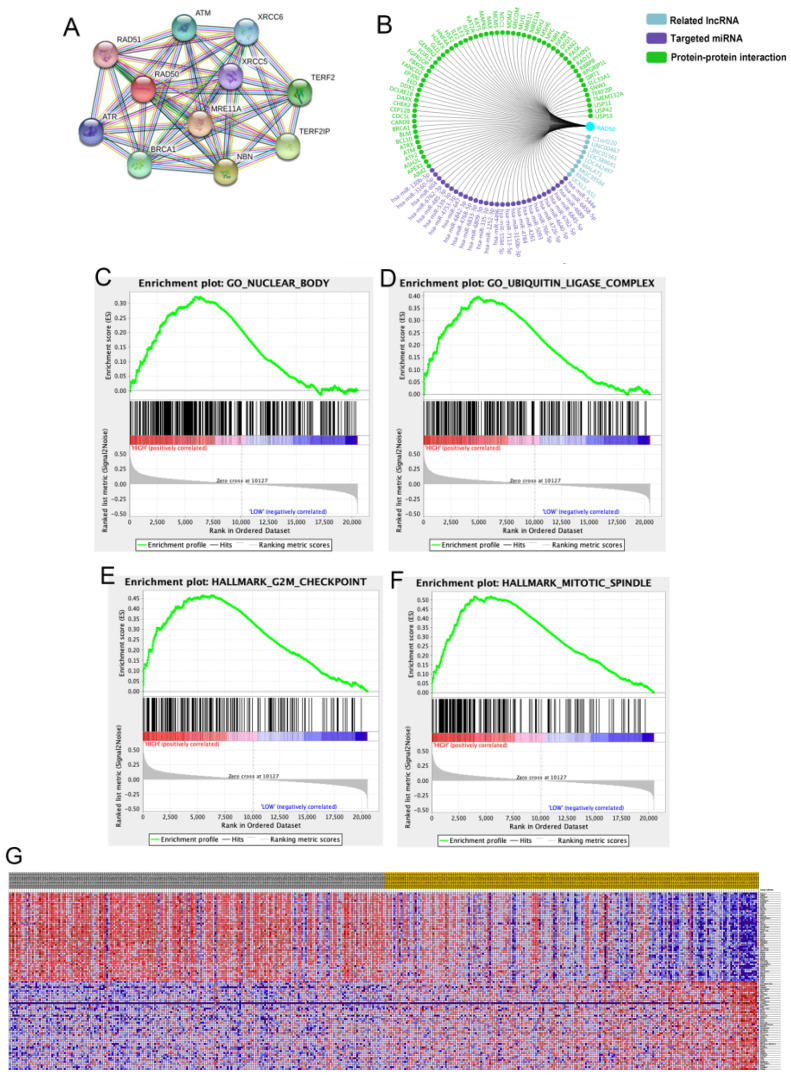
RAD50 related protein analysis. **A-B.** A total of 11 significant genes, including RAD51, ATM, XRCC6, RAD50, XRCC5, TERF2, ATR, MRE11A, BRAC1, NBN and TERF2IP, were identified in the molecular model. **C-G.** Datasets from the TCGA database were analysed with the GSEA method.

A total of 11 significant genes, including *RAD51, ATM, XRCC6, RAD50, XRCC5, TERF2, ATR, MRE11A, BRAC1, NBN* and *TERF2IP*, were included in the molecular model shown in **Fig. 6A**. The significant and potentially related Inc RNAs, targeted miRNAs and protein-protein interaction nodes are shown in **Fig. 6B**. Significant differences were found among the four validation datasets (*p* < 0.05). A total of 100 significant genes were obtained from GSEA, and the genes with positive correlations were plotted. In addition, RAD50 was found to be involved in pathways from most functional annotation categories, including the nuclear body, ubiquitin ligase complex, G2M checkpoint and mitotic spindle signalling pathways. The details are shown in **Fig. 6**.

### Functional enrichment analysis

**Figure 7:**
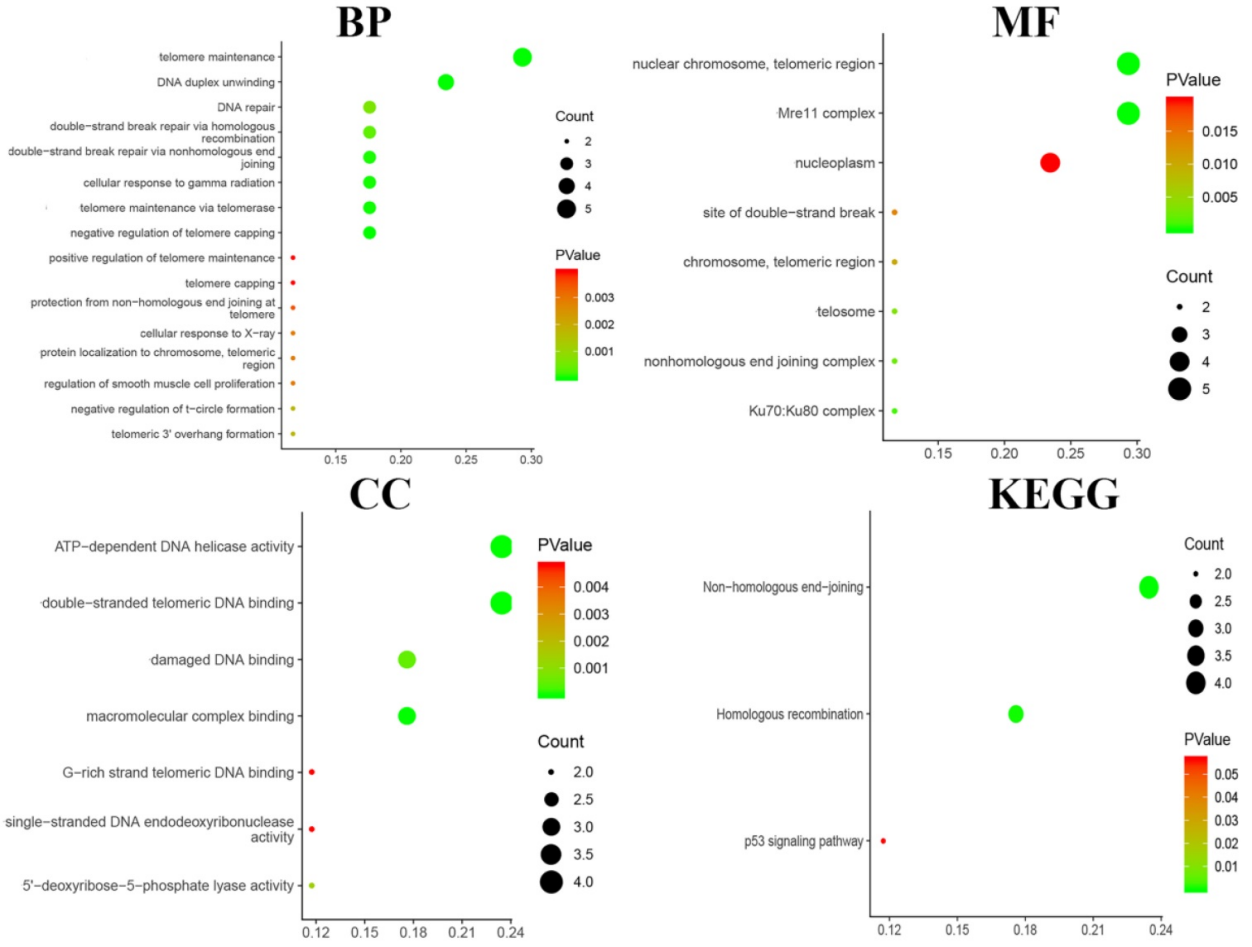
Functional and pathway enrichment analyses were performed using DAVID to obtain a bubble chart. Biological processes, cellular components, molecular functions and the KEGG analysis of the DEGs indicated the most significant signalling pathways.

Functional and pathway enrichment analyses, including analyses of biological processes, cellular components, molecular functions and KEGG pathways, were performed using DAVID and are represented in the bubble chart in **Fig. 7**. The results of GO analyses showed that changes in BPs were significantly enriched in telomere maintenance, DNA duplex unwinding and DNA repair; changes in MFs were mostly enriched in nuclear chromosome and telomeric region; changes in CCs were mostly enriched in ATP-dependent DNA helicase activity and damaged DNA binding. Changes in KEGG pathway enrichment analyses were existed in non-homologous end-joining and homologous recombination.

## Discussion

Herein, we demonstrated that RAD50 was positively correlated with poor prognosis in HCC patients in the TCGA cohort. In the two validated cohorts, increased RAD50 expression was observed in cancer tissues compared to that in para-cancer tissues, which was shown be associated with decreased OS and DFS. Four validation cohorts from the Oncomine database also showed elevated RAD50 expression in HCC tissues compared with that in adjacent normal tissues. Additionally, the functional annotation bioinformatics analysis identified significant related hub genes and signalling pathways.

According to the Int Act molecular interaction database, human RAD50 potentially interacts with the transcription factor c-Jun/activator protein 1 (AP-1) (website: https://www.ebi.ac.uk/intact/interactors/id:Q92878*). C-Jun induces HBV-related liver tumour genes in mice via upregulating the transcription of its target gene osteo pontin (OPN) 17. OPN is overexpressed in several human carcinomas and contributes to inflammation, tumour progression, and metastasis 18, 19. Therefore, we speculated that the interaction between RAD50 and c-Jun promoted the transcription activity of c-Jun, upregulated OPN expression, facilitated the development of HBV-related HCC, and contributed to poor prognosis.

Our study suggested that increased RAD50 expression in HBV-related HCC is a marker of poor prognosis. The researchers have designed a chemical forward genetic screen to identify a small-molecule inhibitor of the MRN complex, named Z-5-(4-hydroxybenzylidene)-2-imino-1, 3-thiazolidin-4-one (mirin) 20. In 2009, other researchers have confirmed the structure of mirin 21. However, there have been no reports about the applications of mirin in cellular, animal and clinical levels. We questioned whether mirin could be used in an HBV-related HCC animal model to determine the therapeutic effect via the inhibition of the MRN complex.

The predictive power of RAD50 may be as strong as that of the classical factors commonly used in clinical practice. Serum tumour markers are the most commonly used markers for the monitoring and early diagnosis of HBV-related HCC because they are non-invasive, objective, and reproducible. The most common serological marker is AFP. Disappointingly, in a retrospective case-control study, the sensitivity of AFP test for the most effective cut-off value (10-20 ng/ml) in the diagnosis of HCC was approximately 60%, and its specificity was 80% 22, 23. These values are much lower than those required for the treatment and prediction of prognosis of HCC patients. However, RAD50 was shown to be a significant and independent biomarker 24. The data indicate that RAD50 may be used to shed further light on the mechanisms underlying HCC progression, to facilitate the discovery of new therapies, and to open up new avenues for the personalized treatment of HCC patients.

In the study, the analysis of the data from the two cohorts supported our hypothesis and clearly demonstrated the high expression of RAD50 in tumour tissues from HCC patients, which results in increases in the HCC recurrence rate and poor overall survival. Furthermore, GSEA indicated that RAD50 is involved in some of the most important pathways, including the mitotic spindle, ultraviolet (UV) response and transforming growth factor beta (TGF-β) signalling pathways that are enriched in HCC specimens. In addition to participating in the DSB repair pathway, RAD50 also interacts with WD repeat and coiled coil containing (MMAP), which is expressed by the mitotic-specific MRN complex to maintain optimal genomic stability during mitosis 25. The question whether RAD50 contributes to HBV-related HCC progression via interacting with MMAP needs further investigation.

The relationship between RAD50 and HCC has rarely been investigated 26. However, it is worth noting that RAD50 is a repair factor that has been identified in the DSB reaction and is highly expressed in many cancers. With this in mind, to further explain the role of RAD50 in the invasion and metastasis of HCC, we used GSEA to analyse data from public databases to identify important genes and pathways that may be involved in the relevance of RAD50 to the initiation of carcinogenesis.

The limitations of this study are described as follows. First, the retrospective nature of the data set is obvious. Although data was obtained from AHYMUN and AHNTU cohorts, the sample size is relatively small, and the patients in the two Chinese cohorts exhibit economic and geographical differences, which may lead differences in therapeutic strategies and overall prognoses. Second, while our research involved a thorough functional annotation of RAD50 in HBV-related HCC, we failed to confirm the underlying mechanisms. In the future, we will conduct multi-centre research in Europe and the United States to further explore the role of RAD50 in the development and prognosis of HCC. RAD50 shows as an indicator of poor prognosis in HBV-related HCC, suggesting a potential target for the therapy of HBV-related HCC.

## Conclusion

Our study reveals that elevated RAD50 expression is significantly correlated with cancer progression and poor survival in HBV-related HCC patients. These data suggest that RAD50, which is a potential therapeutic target, may act as an oncoprotein and could serve as a promising prognostic marker in HCC patients. In this regard, randomized clinical trials and further investigation are required to identify the true value of RAD50 in terms of its clinical application in HCC patients.

## Supplementary Material

Supplementary figure S1.

## Figures and Tables

**Table 1:** Clinicopathological characteristics in relation to RAD50 expression status in two cohorts were shown

Characteristics N (%)	AHYMUM cohort (N=107)	RAD50 expression		χ^2^	*P*	AHNTU cohort (N=100)	RAD50 expression		χ^2^	*P*
		Low IHC score (N=53)	High IHC score (N=54)				Low IHC score (N=52)	High IHC score (N=48)		
**Age**				5.287	**0.021**				0.038	0.844
<60 years	85 (79.4)	40 (75.5)	45 (83.3)			72 (72.0)	37 (71.2)	35 (72.9)		
≥60 years	22 (20.6)	13 (24.5)	9 (16.7)			28 (28.0)	15 (28.2)	13 (27.1)		
**Gender**				0.698	0.403				1.512	0.219
Male	90 (84.1)	43 (81.1)	47 (87.0)			82 (82.0)	45 (86.5)	37 (77.1)		
Female	17 (15.9)	10 (18.9)	7 (13.0)			18 (18.0)	7 (13.5)	11 (22.9)		
**Tumor size**				3.447	0.063				13.117	**<0.001**
<5 cm	59 (55.1)	34 (64.2)	25 (46.3)			48 (48.0)	34 (65.4)	14 (29.2)		
≥5 cm	48 (44.9)	19 (35.8)	29 (53.7)			52 (52.0)	18 (34.6)	34 (70.8)		
**AJCC stage**				10.363	**0.001**				9.324	**0.003**
I - II	94 (87.9)	52 (98.1)	42 (77.8)			66 (66.0)	42 (80.8)	14 (29.2)		
III - IV	13 (12.1)	1 (1.9)	12 (22.2)			34 (34.0)	10 (19.2)	24 (70.8)		
**Primary lesion**				10.194	**0.001**				6.732	**0.010**
Single	83 (77.8)	48 (90.6)	35 (64.8)			79 (79.0)	46 (88.5)	33 (68.8)		
≥2	24 (22.4)	5 (9.4)	19 (35.2)			21 (21.0)	6 (11.5)	15 (31.2)		
**Albumin level**				0.291	0.590				0.496	0.503
<35 g/L	37 (34.6)	17 (32.1)	20 (37.0)			51 (51.0)	23 (44.2)	28 (58.3)		
≥35 g/L	70 (65.4)	36 (67.9)	34 (63.0)			49 (49.0)	29 (55.8)	20 (41.7)		
**Bilirubin level**				0.641	0.423				1.803	0.179
<20.4 umol/L	77 (72.0)	40 (75.5)	37 (68.5)			65 (65.0)	37 (71.2)	28 (58.3)		
≥20.4 umol /L	30 (28.0)	13 (24.5)	17 (31.5)			35 (35.0)	15 (28.8)	20 (41.7)		
**Liver cirrhosis**				2.905	0.088				0.550	0.458
Absent	6 (5.6)	5 (9.4)	1 (1.9)			6 (6.0)	4 (7.7)	2 (4.2)		
Present	101 (94.4)	48 (90.6)	53 (98.1)			94 (94.0)	48 (92.3)	46 (95.8)		
**Differential degree**				0.849	0.357				7.771	**0.005**
Low	30 (28.0)	17 (32.1)	13 (24.1)			62 (62.0)	39 (75.0)	23 (47.9)		
High	77 (72.0)	36 (67.9)	41 (75.9)			38 (38.0)	13 (25.0)	25 (52.1)		
**Microvascular invasion**				4.821	**0.028**				10.359	**0.001**
Absent	96 (89.7)	51 (96.2)	45 (83.3)			62 (62.0)	40 (88.9)	22 (45.8)		
Present	11 (10.3)	2 (3.8)	9 (16.7)			38 (38.0)	12 (11.1)	26 (54.2)		
**Capsular invasion**				5.922	**0.015**				9.324	**0.003**
Absent	87 (81.3)	48 (90.6)	39 (72.2)			67 (67.0)	42 (80.8)	25 (52.1)		
Present	20 (18.7)	5 (9.4)	15 (27.8)			33 (33.0)	10 (19.2)	23 (47.9)		
**Okuda score**				6.805	**0.009**				7.015	**0.008**
I	51 (47.7)	32 (60.4)	19 (35.2)			69 (69.0)	42 (80.8)	27 (56.3)		
II	56 (52.3)	21 (39.6)	35 (64.8)			31 (31.0)	10 (19.2)	21 (43.8)		

**Table 2:** Multivariate Cox regression analysis of DFS in AHYMUM and AHNTU cohorts (DFS: disease-free survival) was shown

Covariates	AHYMUM			AHNTU		
	HR	95% CI	*P* value	HR	95% CI	*P* value
Tumor size (ref. <5cm)	-	-	-	0.839	0.412-1.707	0.627
AJCC stage (ref. II - IV)	0.660	0.267-1.631	0.269	1.796	0.912-3.538	0.090
Primary lesion (ref. Single)	1.156	0.706-2.349	0.155	2.563	1.286-5.110	**0.007**
Differential degree (ref. Low)	2.052	0.964-4.371	0.062	1.404	0.633-3.110	0.404
Microvascular invasion (ref. Absent)	1.020	0.374-2.780	0.969	1.317	0.626-2.769	0.468
Capsular invasion (ref. Absent)	1.281	0.672-2.441	1.281	0.899	0.418-1.933	0.784
Okuda score (ref. I)	1.914	1.014-3.611	**0.045**	3.084	1.632-5.828	**0.001**
RAD50 expression (ref. low)	4.612	2.336-9.106	**<0.001**	2.660	1.331-5.317	**0.006**

**Table 3:** Multivariate Cox regression analysis of OS in AHYMUM and AHNTU cohorts (OS: overall survival) was shown

Covariates	AHYMUM			AHNTU		
	HR	95% CI	*P* value	HR	95% CI	*P* value
Tumor size (ref. <5cm)	1.199	0.463-3.104	0.708	0.905	0.358-2.283	0.832
AJCC stage (ref. II - IV)	0.763	0.256-2.277	0.628	2.826	1.150-6.947	**0.024**
Primary lesion (ref. Single)	1.151	0.514-0.576	0.733	2.151	0.898-5.154	0.086
Differential degree (ref. Low)	2.019	0.792-5.147	0.141	0.925	0.377-2.269	0.865
Microvascular invasion (ref. Absent)	1.488	0.719-3.081	0.284	2.023	0.783-5.228	0.146
Capsular invasion (ref. Absent)	0.984	0.326-2.973	0.978	1.363	0.518-3.585	0.530
Okuda score (ref. I)	2.329	1.079-5.027	**0.031**	2.819	1.105-7.194	**0.030**
RAD50 expression (ref. low)	6.807	2.598-17.839	**<0.001**	1.735	0.753-3.996	0.195

## Abbreviations

AHNTU: Affiliated Hospital of Nantong University
AHYMUM: Affiliated Hospital of YouJiang Medical University for Nationalities
BP: biological process
CC: cellular component
CI: confidence interval
DD: DNA damage response
DFS: disease-free survival
GO: gene ontology
HBV: hepatitis B virus
HCC: Hepatocellular carcinoma
HR: hazard ratio
IHC: immunohistochemistry
KEGG: Kyoto Encyclopedia of Genes and Genomes
MF: molecular function
OS: overall survival
TCGA: the Cancer Genome Atlas
