# Supplementary figures and images for: Elevated double-strand break repair protein RAD50 predicts poor prognosis in hepatitis B virus-related hepatocellular carcinoma: A study based on Chinese high-risk cohorts
Source: J Cancer. 2020 Aug 14;11(20):5941–52. doi: 10.7150/jca.46703 (PMC7477405; doi:10.7150/jca.46703)

**Figure S1: Fig. S1.** Different intensity of RAD50 immunostaining expression.

A: 0-25%, B: 26-50%, C: 51-75%, D: 76-100%.

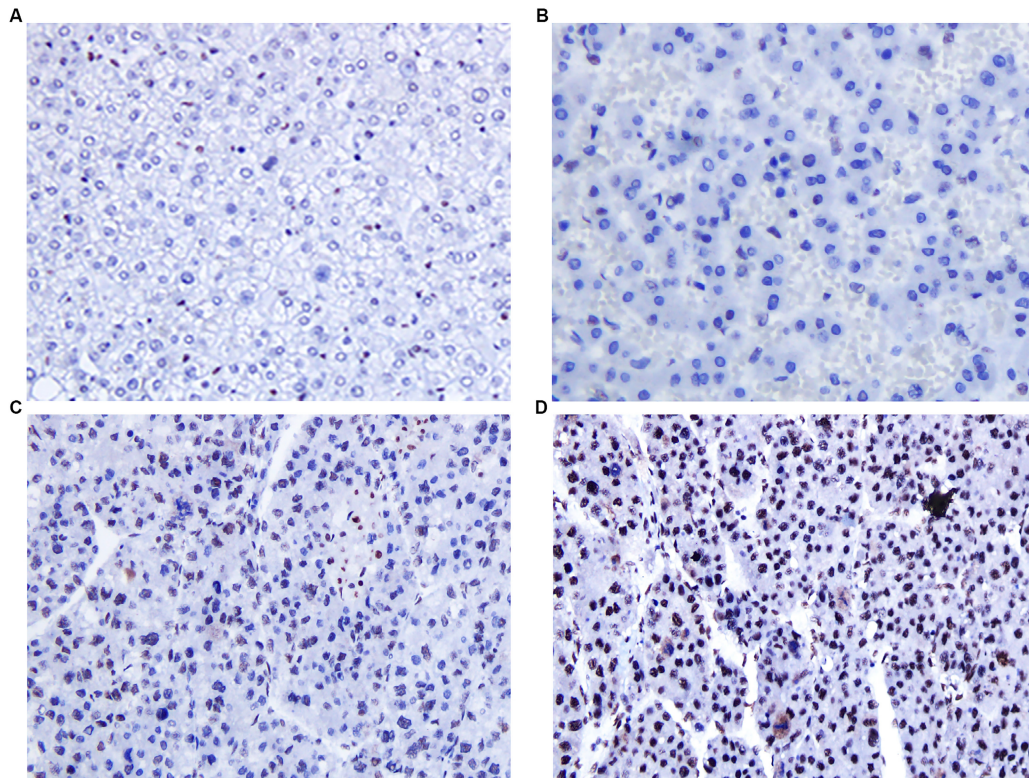

Supplement: Supplementary file 1 — Supplementary figure S1. [file jcav11p5941s1.pdf]
